# Supplementary material for: The genomic landscape of pediatric myelodysplastic syndromes
Source: Nat Commun. 2017 Nov 16;8:1557. doi: 10.1038/s41467-017-01590-5 (PMC5691144; doi:10.1038/s41467-017-01590-5)
Supplement: Supplementary file 1 — Supplementary Information [file 41467_2017_1590_MOESM1_ESM.pdf]

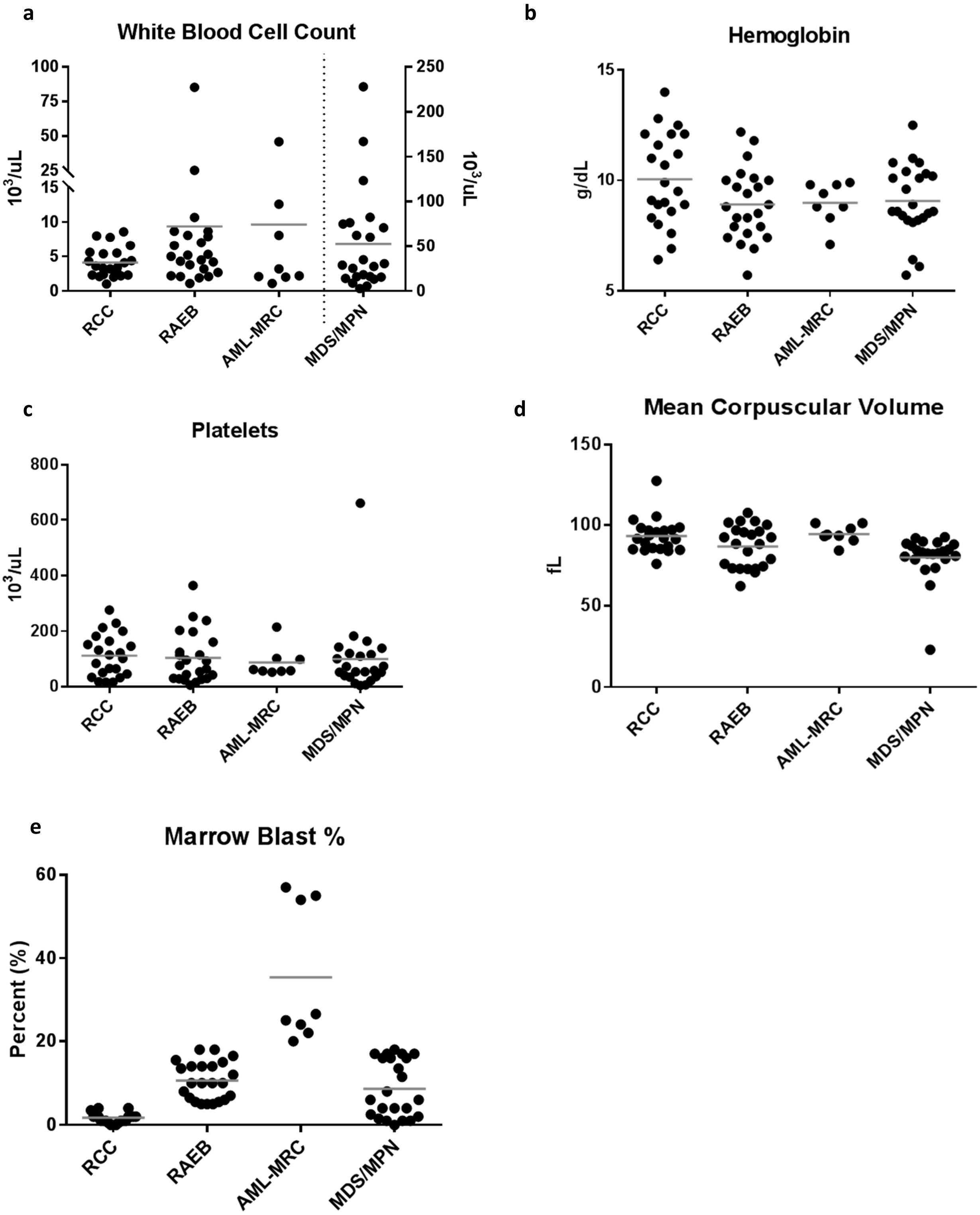

Supplementary Figure 1: a-d.) CBC values by subtype. e.) Bone marrow blast % at the time of diagnosis by subtype.

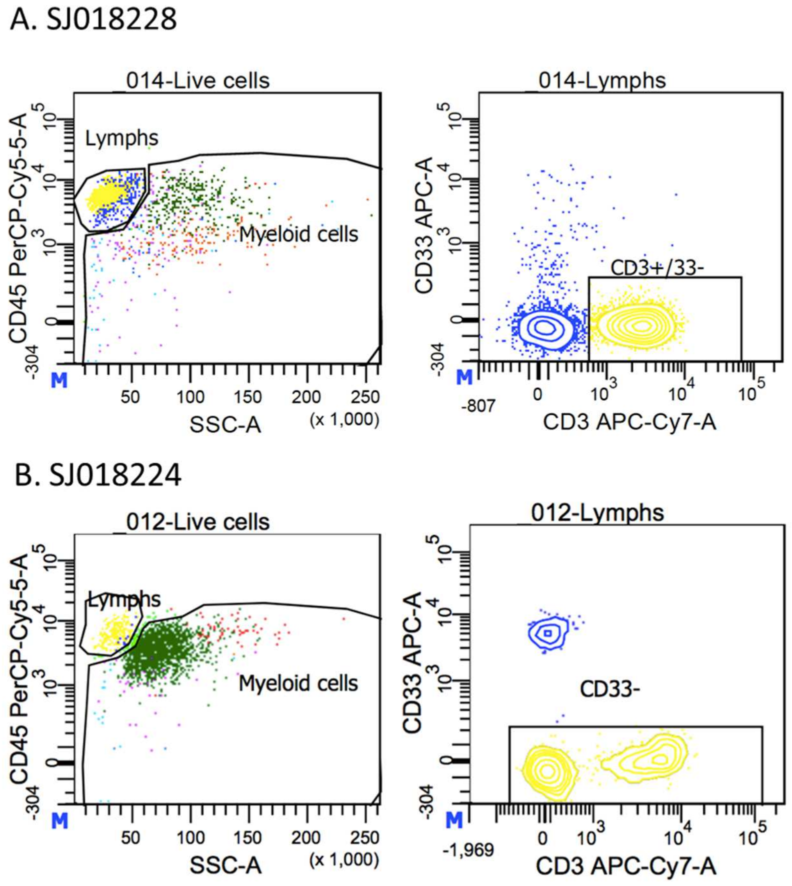

Supplementary Figure 2: Flow sorting strategy to collect normal and tumor gDNA. Two (A & B) representative examples of pediatric MDS demonstrating the separation of live cells (via DAPI exclusion) by CD45 expression and side scatter characteristics (left). Contaminating myeloid cells in the lymphocyte gate were excluded using CD33 expression. When sufficient numbers were available, we collected only CD3+ T-cells (top). If not, we collected all CD33^{neg} events in the lymphocyte gate (bottom). Myeloid cells were collected based on side scatter and CD45 expression.

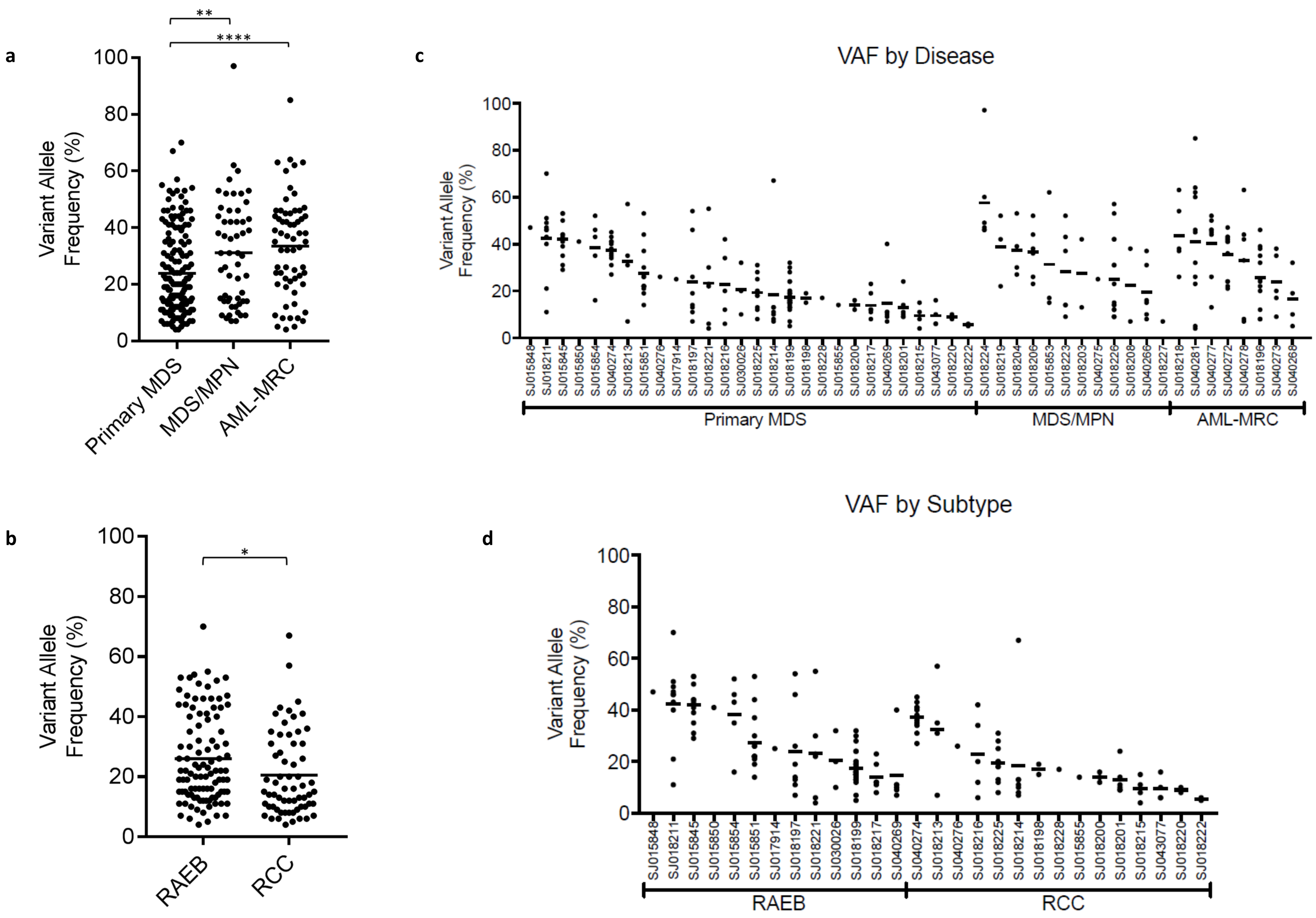
a
**
****
100
80
60
40
20
0
Variant Allele Frequency (%)
Primary MDS
MDS/MPN
AML-MRC
b
*
100
80
60
40
20
0
Variant Allele Frequency (%)
RAEB
RCC
c
VAF by Disease
100
80
60
40
20
0
Variant Allele Frequency (%)
SJ015848
SJ018211
SJ015845
SJ015850
SJ015854
SJ040274
SJ018213
SJ015851
SJ040276
SJ017914
SJ018197
SJ018221
SJ018216
SJ030026
SJ018225
SJ018214
SJ018199
SJ018198
SJ018228
SJ015855
SJ018200
SJ018217
SJ040269
SJ018201
SJ018215
SJ043077
SJ018220
SJ018222
SJ018224
SJ018219
SJ018204
SJ018206
SJ015853
SJ018223
SJ018203
SJ040275
SJ018226
SJ018208
SJ040266
SJ018227
SJ018218
SJ040281
SJ040277
SJ040272
SJ040278
SJ018196
SJ040273
SJ040268
Primary MDS
MDS/MPN
AML-MRC
d
VAF by Subtype
100
80
60
40
20
0
Variant Allele Frequency (%)
SJ015848
SJ018211
SJ015845
SJ015850
SJ015854
SJ015851
SJ017914
SJ018197
SJ018221
SJ030026
SJ018199
SJ018217
SJ040269
SJ040274
SJ018213
SJ040276
SJ018216
SJ018225
SJ018214
SJ018198
SJ018228
SJ015855
SJ018200
SJ018201
SJ018215
SJ043077
SJ018220
SJ018222
RAEB
RCC

Supplementary Figure 3: Somatic mutation VAF's determined by WES. a.) All somatic mutation VAF's categorized by diagnosis (n=54). **: $p<0.01$; ****: $p<0.0001$ (student's t-test). b.) All somatic mutation VAF's within the primary MDS cohort categorized by disease subtype (n=32). *$p<0.05$ (student's t-test). c.) All somatic mutation VAF's shown per patient. d.) All somatic mutation VAF's within the primary MDS cohort shown per patient. Six patients are not shown given the lack of somatic mutations (RCC: 3; RAEB: 1; MDS/MPN: 2).

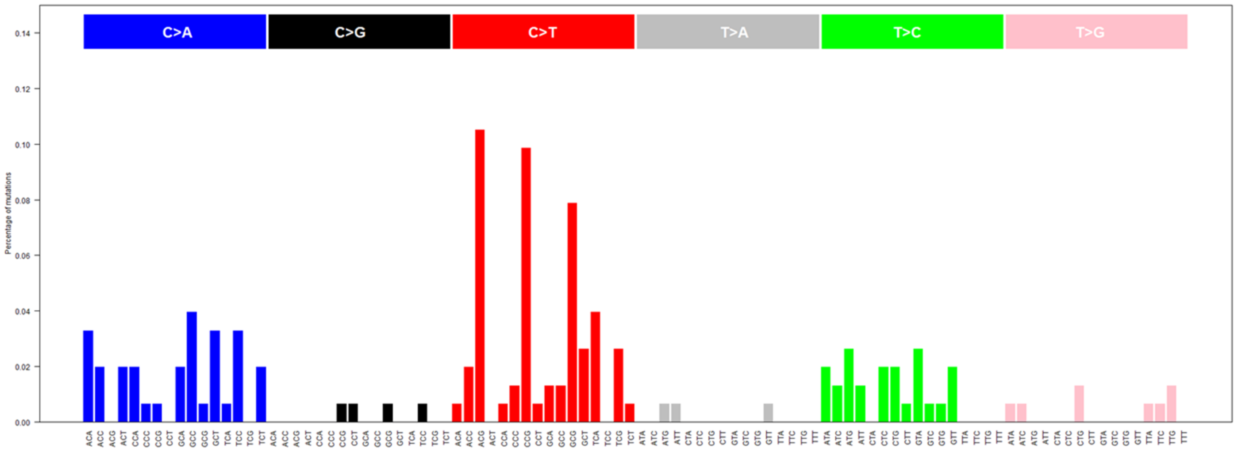

Supplementary Figure 4: Mutational signature of pediatric primary MDS. The primary MDS mutational signature, characterized by C>T substitutions at NpCpG trinucleotides, is similar to the Signature 1A/1B described in AML[1]. We used the published computational framework for mutation signature identification (http://www.mathworks.com/matlabcentral/fileexchange/38724). The algorithm deciphers the minimal set of mutational signatures that optimally explains the proportion of each mutation type found in each catalogue and then estimates the contribution of each signature to each catalogue. We used high-quality somatic mutations called from the whole exome sequencing samples of primary MDS (28 samples, 152 mutations).

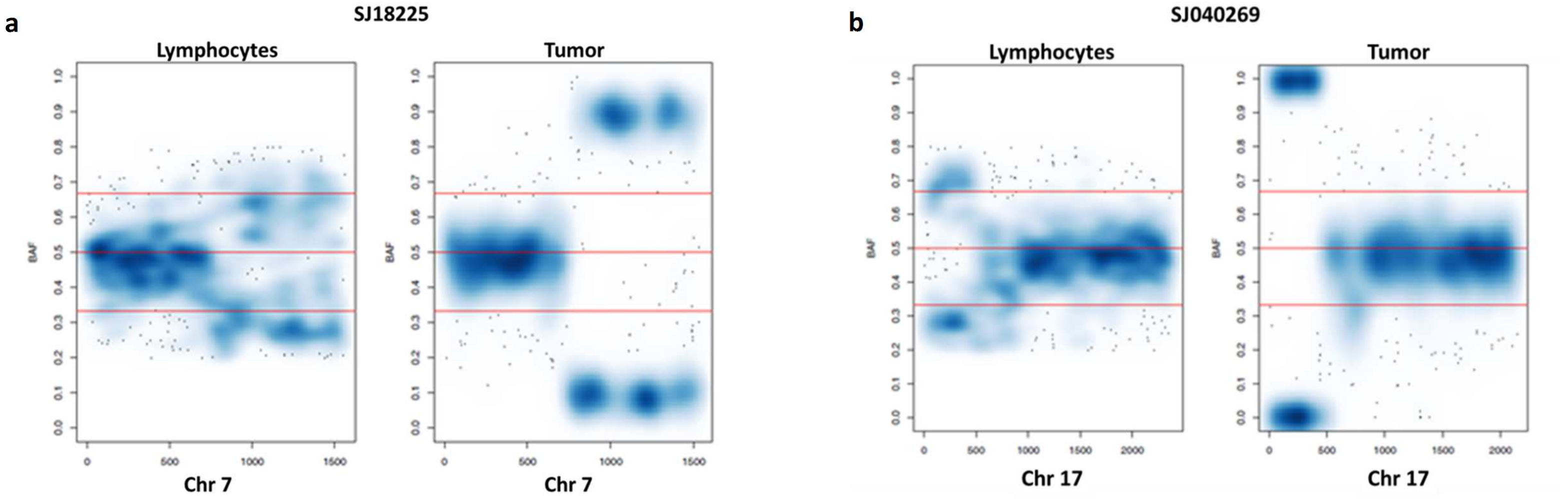

Supplementary Figure 5: Copy number alterations found in the pediatric MDS cohort in the lymphocyte samples. Three cases in the exome cohort showed evidence of subclonal CN-LOH events in the lymphocyte exome data. Considering the subclonal nature, we favor these to be acquired events, rather than germline. a.) Allele frequency plot of an RCC patient with subclonal chromosome 7q CN-LOH in the lymphocytes with a clonal chromosome 7 CN-LOH in the MDS tumor population. This CN-LOH region includes the *SAMD9/SAMD9L* locus in a patient with a germline *SAMD9L* mutation. We also previously reported the CN-LOH involving 7q in SJ018228 that involves the *SAMD9/SAMD9L* locus[2] (Data not shown here). b.) A RAEB patient with chromosome 17p CN-LOH, that includes *TP53*. In the MDS tumor cells, there appears to be a clonal event.

a

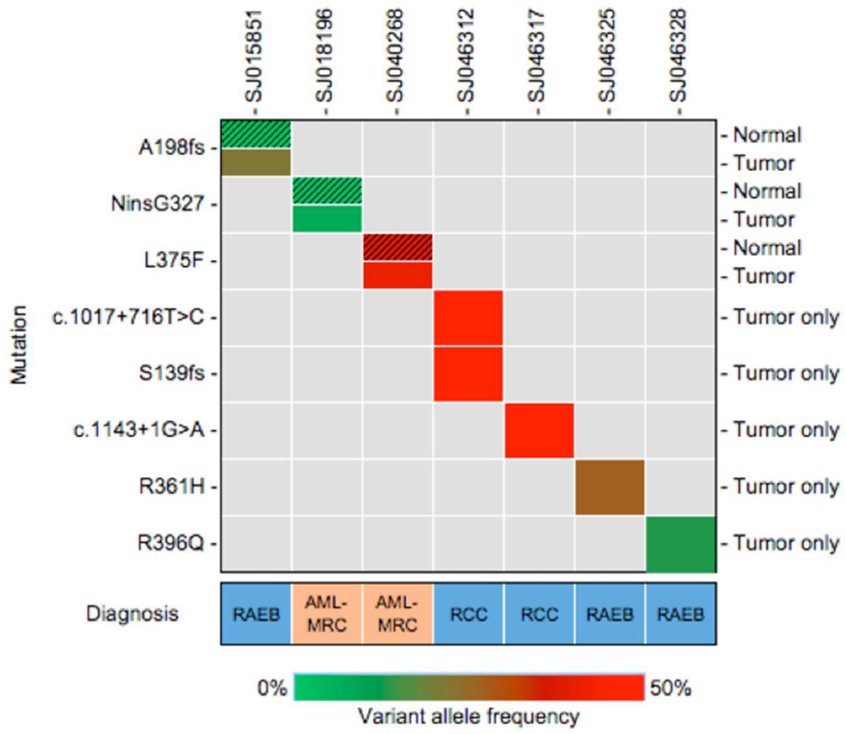

b

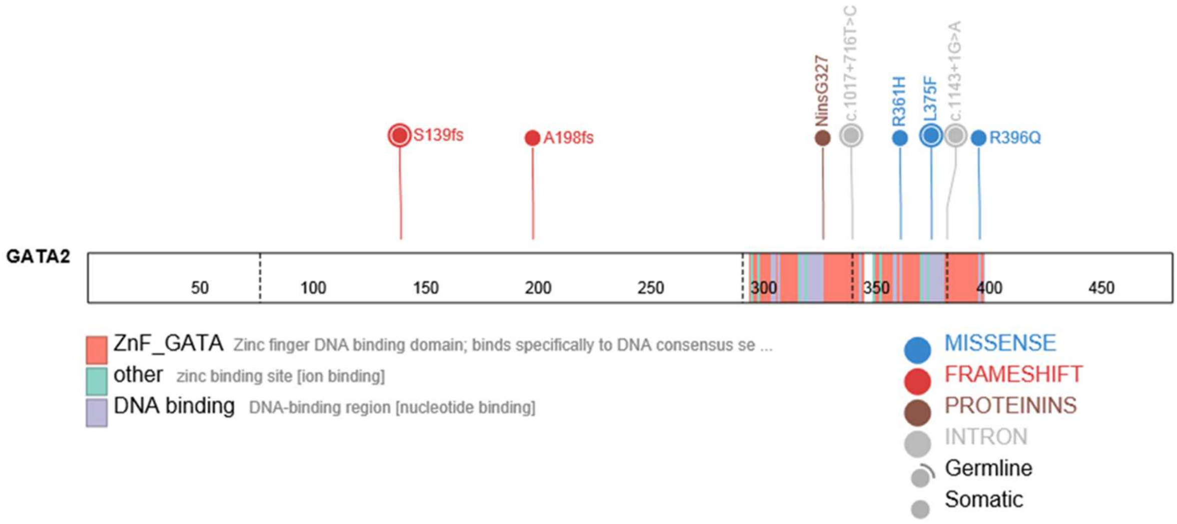

Supplementary Figure 6: *GATA2* mutations present in the pediatric MDS cohort. a.) Chart showing cases with a *GATA2* mutation. Right column indicates in which source the mutation was identified. Color range is relative to the VAF of the specific mutation. b.) Mutation locations relative to protein domains of *GATA2*.

a

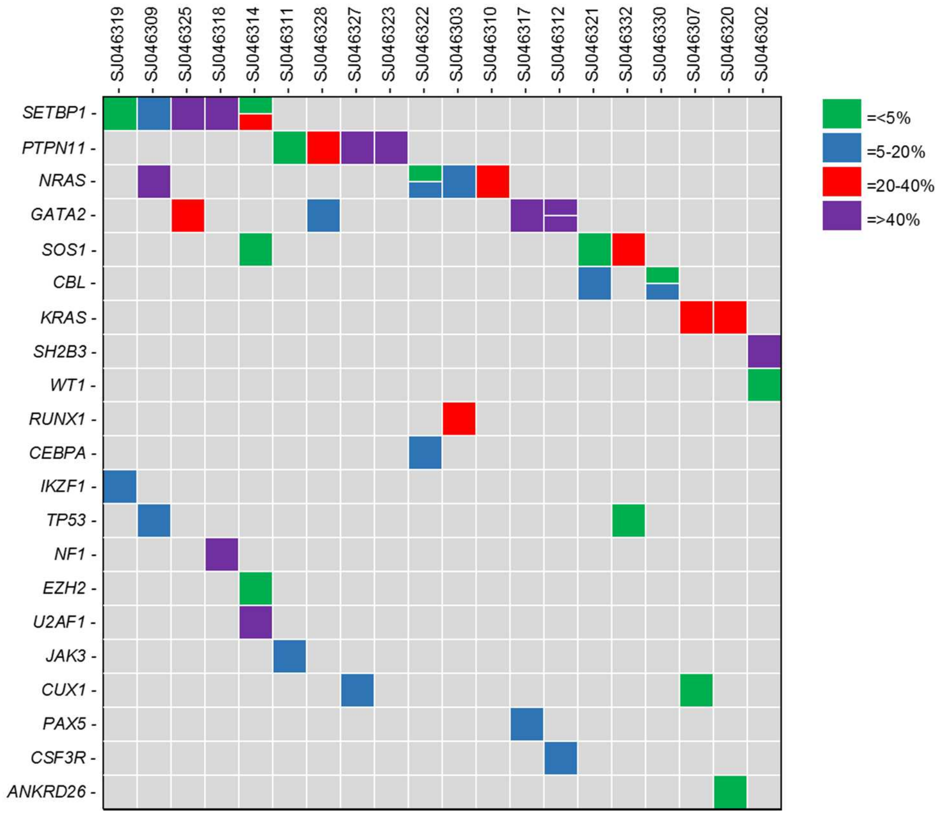

b

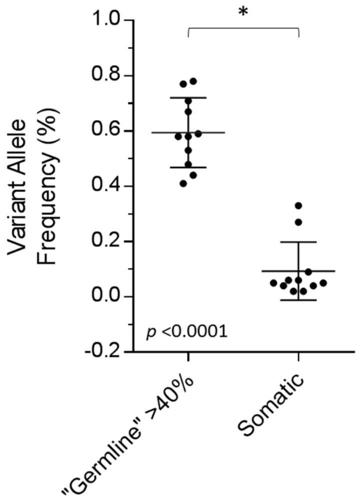

Supplementary Figure 7: a.) Genomic alterations identified in 20 tumor-only cases. Three of the 23 total tumor-only samples had no mutations identified. VAF is indicated by color (see legend). b.) Eleven "germline" variants with VAF's >40% are significantly higher than other somatic variants found in those patients. Error bars represent standard deviation and the means are represented with the center bars.

a

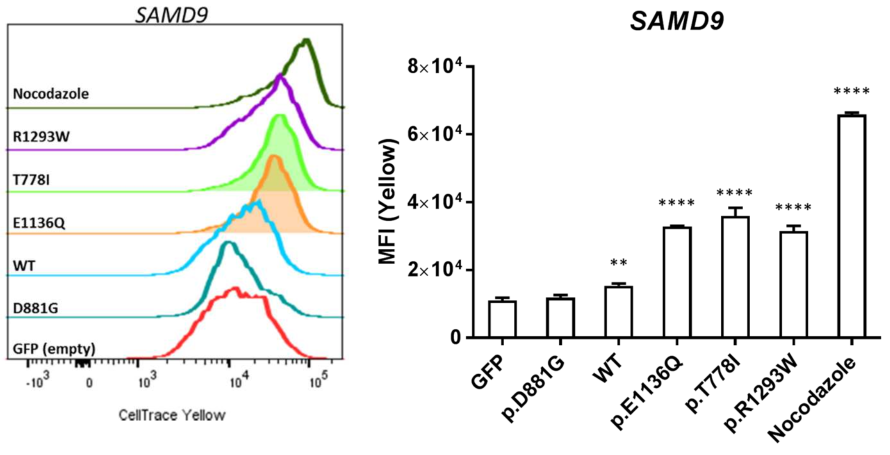
SAMD9
Nocodazole
R1293W
T778I
E1136Q
WT
D881G
GFP (empty)
CellTrace Yellow
SAMD9
MFI (Yellow)
GFP
p.D881G
WT
p.E1136Q
p.T778I
p.R1293W
Nocodazole

b

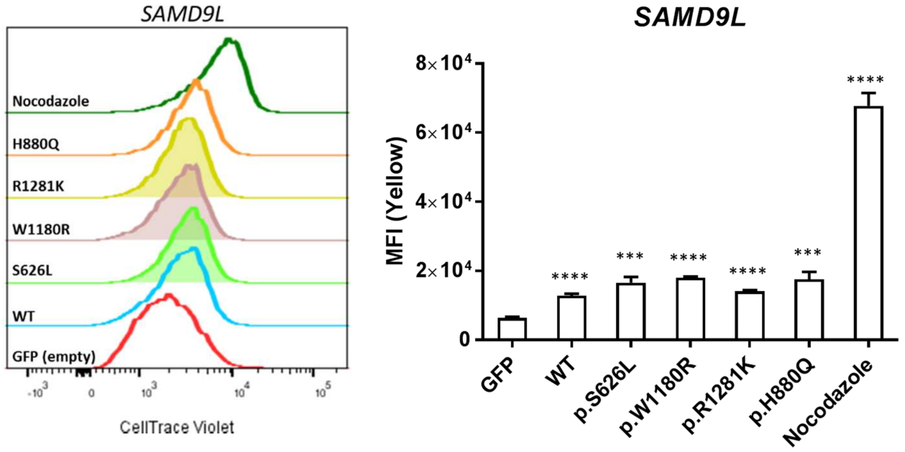
SAMD9L
Nocodazole
H880Q
R1281K
W1180R
S626L
WT
GFP (empty)
CellTrace Violet
SAMD9L
MFI (Yellow)
GFP
WT
p.S626L
p.W1180R
p.R1281K
p.H880Q
Nocodazole

c

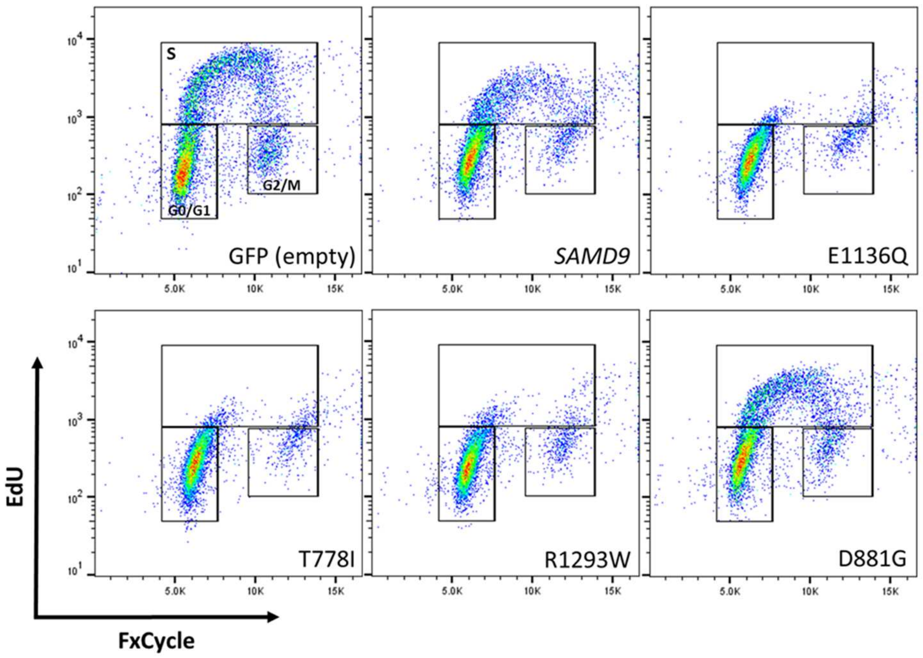
S
G0/G1
G2/M
GFP (empty)
SAMD9
E1136Q
T778I
R1293W
D881G
EdU
FxCycle

Supplementary Figure 8: Gain-of-function mutations in *SAMD9* and *SAMD9L* decrease cell proliferation a.) CellTrace Yellow™ (CTY) dye dilution cell proliferation assay. Nocodazole (control) inhibits microtubule formation and therefore cell proliferation. *SAMD9* p.R1293W (positive control) has previously been shown to be a gain-of-function mutation in MIRAGE syndrome[3]. *SAMD9* p.D881G is a common SNP that is not predicted to be pathogenic. b.) *SAMD9L* CTY dye dilution cell proliferation assay. *SAMD9L* p.H880Q (positive control) is a gain-of-function mutation previously reported in Ataxia-Pancytopenia syndrome[4]. Data is representative of 2-3 experiments completed in triplicate. **: $p<0.01$, ***: $p<0.001$, ****: $p<0.0001$. Error bars indicated standard deviation. c.) Flow cytometry plots (FxCycle-total DNA content vs. EdU incorporation) showing the cell cycle inhibiting effects of gain-of-function *SAMD9* mutations.

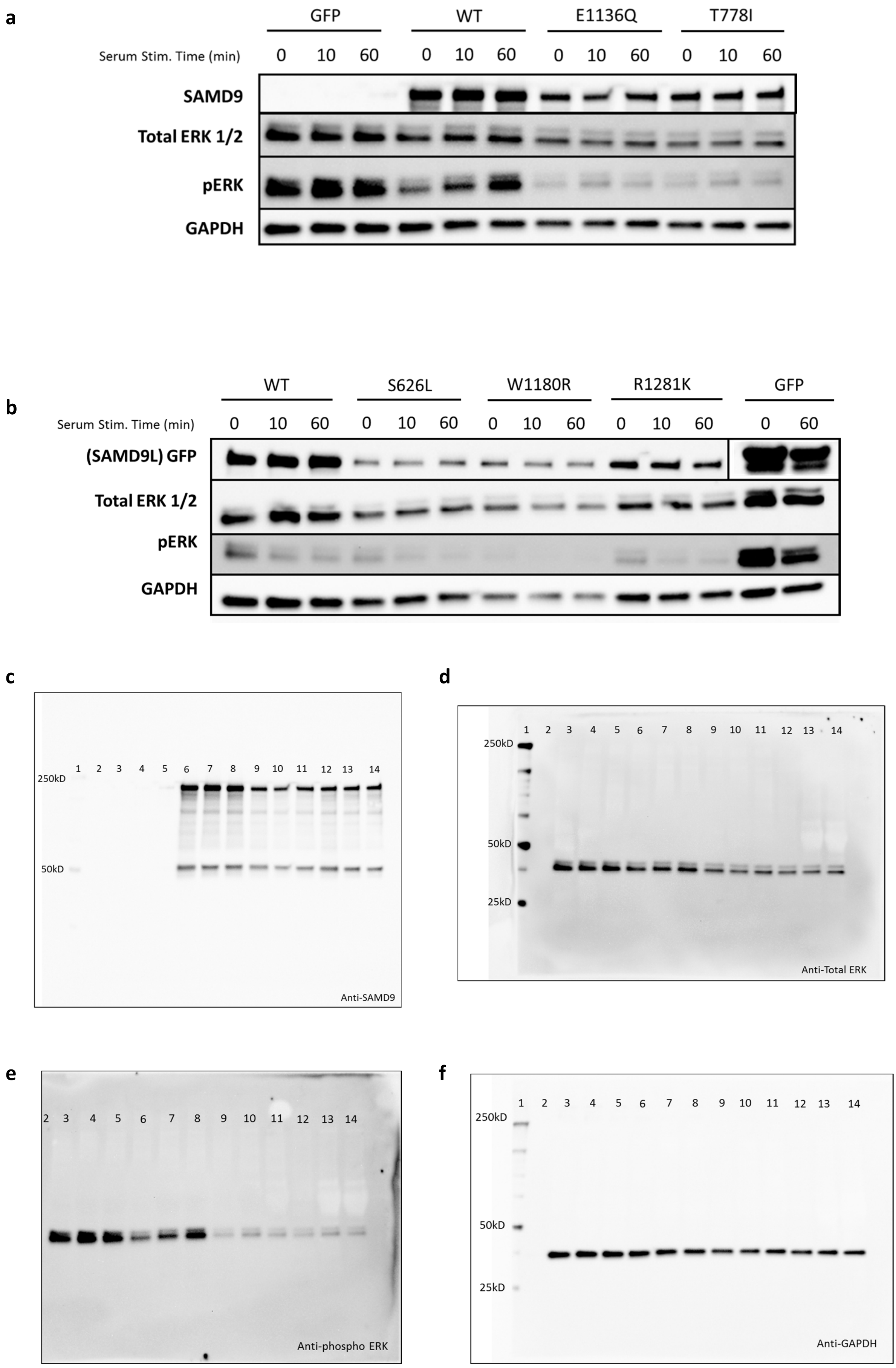
a
GFP
WT
E1136Q
T778I
Serum Stim. Time (min)
0 10 60 0 10 60 0 10 60 0 10 60
SAMD9
Total ERK 1/2
pERK
GAPDH
b
WT
S626L
W1180R
R1281K
GFP
Serum Stim. Time (min)
0 10 60 0 10 60 0 10 60 0 10 60 0 60
(SAMD9L) GFP
Total ERK 1/2
pERK
GAPDH
c
1 2 3 4 5 6 7 8 9 10 11 12 13 14
250kD
50kD
Anti-SAMD9
d
1 2 3 4 5 6 7 8 9 10 11 12 13 14
250kD
50kD
25kD
Anti-Total ERK
e
2 3 4 5 6 7 8 9 10 11 12 13 14
Anti-phospho ERK
f
1 2 3 4 5 6 7 8 9 10 11 12 13 14
250kD
50kD
25kD
Anti-GAPDH

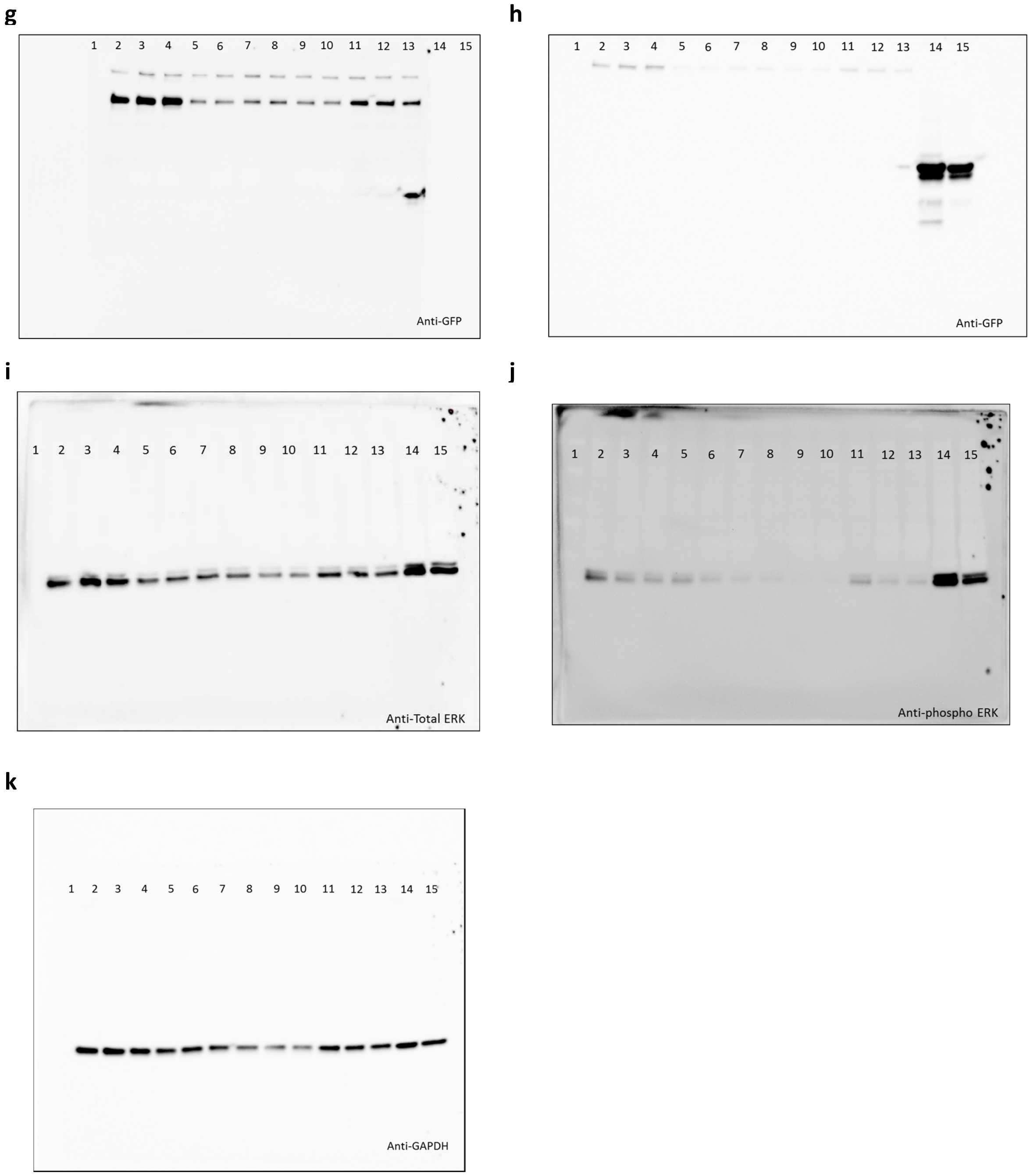

Supplementary Figure 9: SAMD9/L mutations inhibit the induction of ERK phosphorylation. Immunoblots for SAMD9 (a) or GFP (SAMD9L) (b) and phosphorylated ERK following 0, 10, & 60 minutes of serum stimulation. Full western blots of each antibody are shown in panels c (SAMD9), d (Total ERK), e (phosphorylated ERK), and f (GAPDH) for SAMD9 studies. Full western blots of each antibody are shown in panels g & h (GFP), i (Total ERK), j (phosphorylated ERK), and k (GAPDH) for SAMD9L studies. Data is representative of 2 biological replicates. For blots in panels c-f, Lane 1: molecular weight marker, Lane 2: blank, and following lanes correspond to the labels in panel a. For panel e the molecular weight marker was removed from the blot due to substantial differences in chemiluminescence signal between it and the target. For blots in panels g-k Lane 1: molecular weight marker, although the secondary for the marker was not used, therefore the absence of signal. Lanes 2-15 correspond to the labels in panel b. Lanes 14 and 15 of blots in panels g and h were exposed separately given the large differences in chemiluminescence signal.

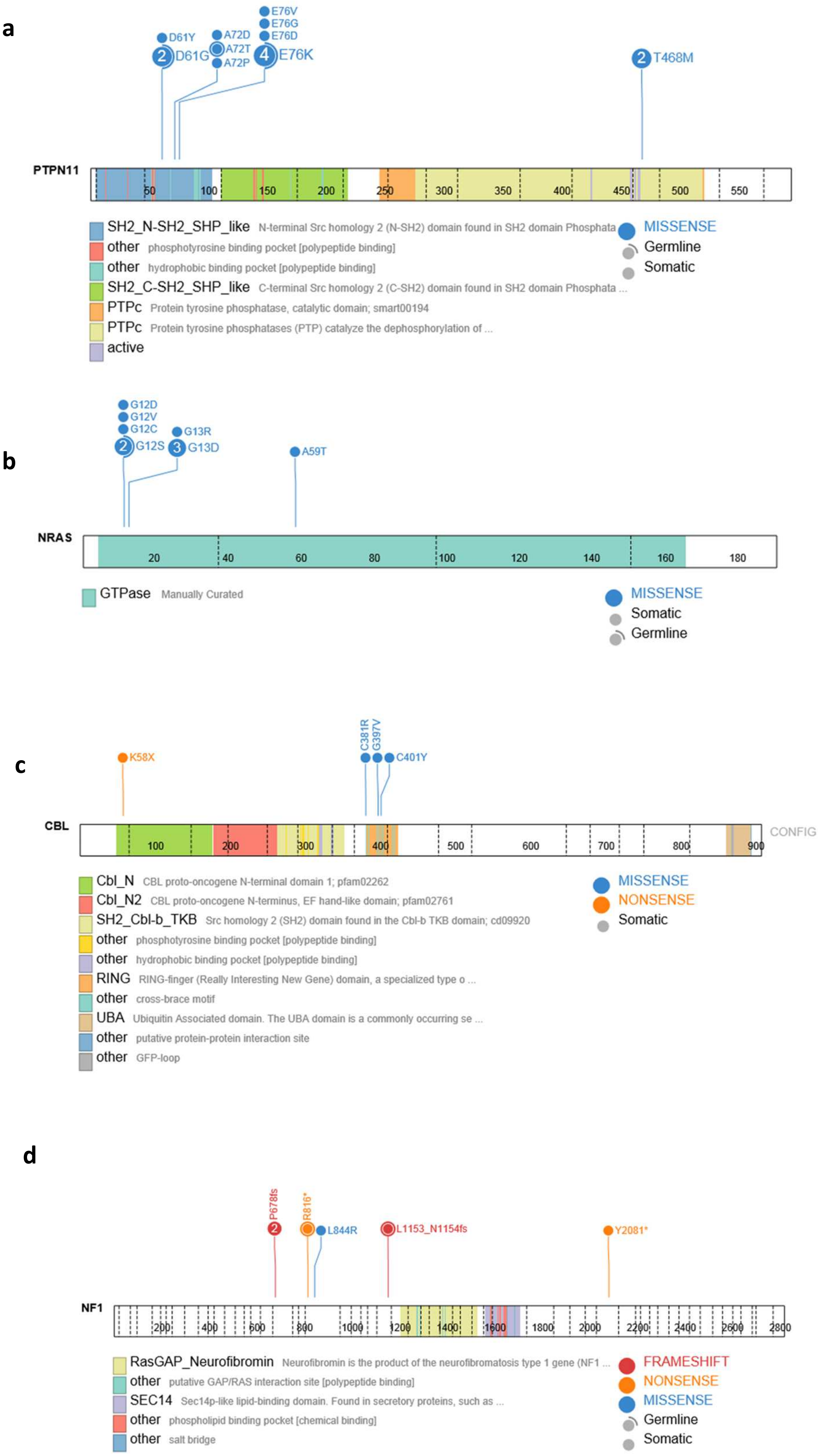
a
D61Y
D61G
A72D
A72T
A72P
E76V
E76G
E76D
E76K
T468M
PTPN11
50
100
150
200
250
300
350
400
450
500
550
SH2_N-SH2_SHP_like N-terminal Src homology 2 (N-SH2) domain found in SH2 domain Phosphata
other phosphotyrosine binding pocket [polypeptide binding]
other hydrophobic binding pocket [polypeptide binding]
SH2_C-SH2_SHP_like C-terminal Src homology 2 (C-SH2) domain found in SH2 domain Phosphata ...
PTPc Protein tyrosine phosphatase, catalytic domain; smart00194
PTPc Protein tyrosine phosphatases (PTP) catalyze the dephosphorylation of ...
active
MISSENSE
Germline
Somatic
b
G12D
G12V
G12C
G12S
G13R
G13D
A59T
NRAS
20
40
60
80
100
120
140
160
180
GTPase Manually Curated
MISSENSE
Somatic
Germline
c
K58X
C381R
G397V
C401Y
CBL
100
200
300
400
500
600
700
800
900
CONFIG
Cbl_N CBL proto-oncogene N-terminal domain 1; pfam02262
Cbl_N2 CBL proto-oncogene N-terminus, EF hand-like domain; pfam02761
SH2_Cbl-b_TKB Src homology 2 (SH2) domain found in the Cbl-b TKB domain; cd09920
other phosphotyrosine binding pocket [polypeptide binding]
other hydrophobic binding pocket [polypeptide binding]
RING RING-finger (Really Interesting New Gene) domain, a specialized type o ...
other cross-brace motif
UBA Ubiquitin Associated domain. The UBA domain is a commonly occurring se ...
other putative protein-protein interaction site
other GFP-loop
MISSENSE
NONSENSE
Somatic
d
P678fs
R816*
L844R
L1153_N1154fs
Y2081*
NF1
200
400
600
800
1000
1200
1400
1600
1800
2000
2200
2400
2600
2800
RasGAP_Neurofibromin Neurofibromin is the product of the neurofibromatosis type 1 gene (NF1 ...
other putative GAP/RAS interaction site [polypeptide binding]
SEC14 Sec14p-like lipid-binding domain. Found in secretory proteins, such as ...
other phospholipid binding pocket [chemical binding]
other salt bridge
FRAMESHIFT
NONSENSE
MISSENSE
Germline
Somatic

e

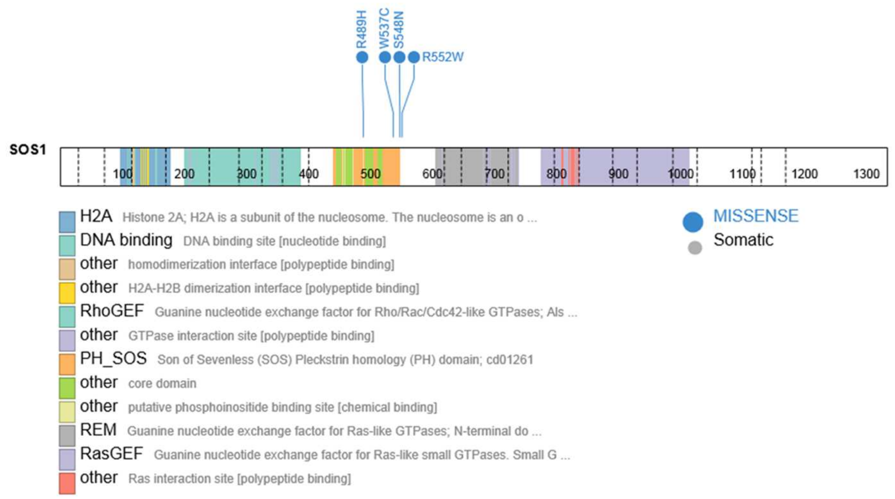

f

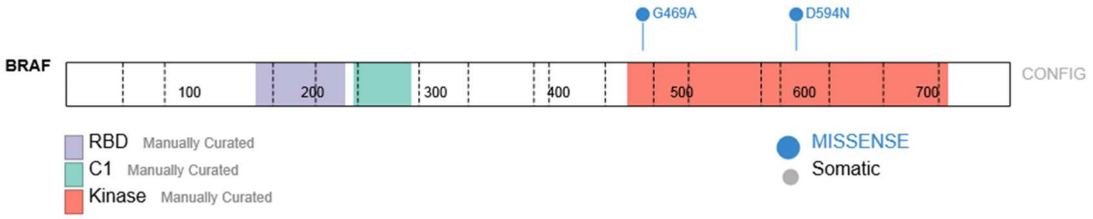

Supplementary Figure 10: Ras/MAPK pathway mutations. Protein Paint schematics showing locations of mutations present in *PTPN11* (a), *NRAS* (b), *CBL* (c), *NF1* (d) *SOS1* (e), *BRAF* (f) found in our pediatric primary MDS cohort.

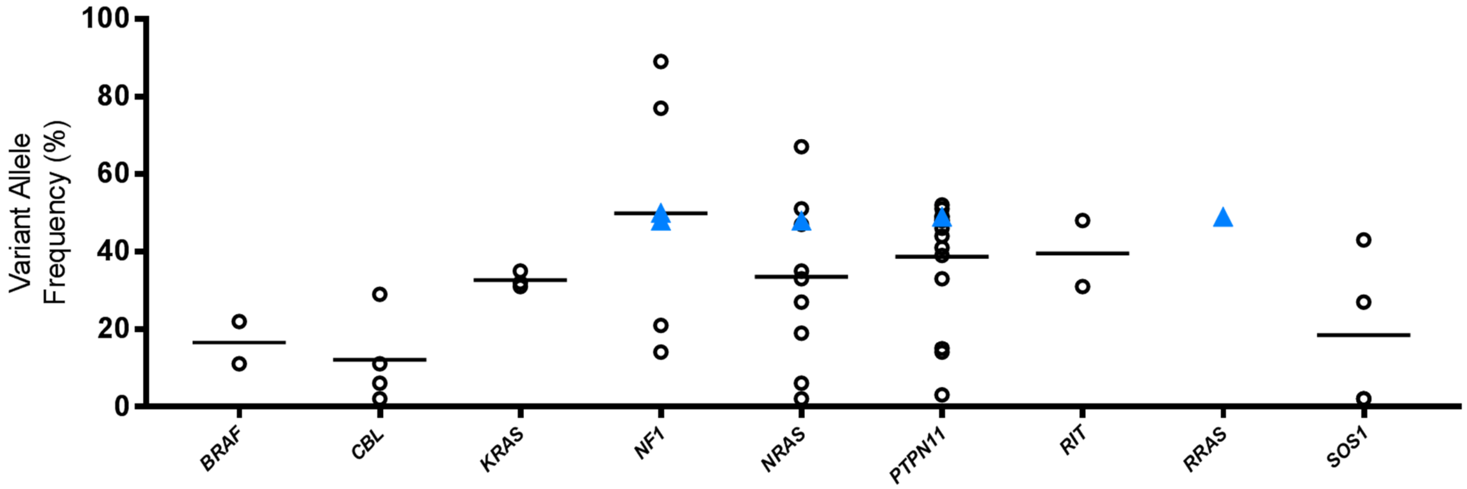

Supplementary Figure 11: Ras/MAPK pathway mutation VAF's. Tumor VAF's of all Ras/MAPK pathway mutations present in the pediatric MDS cohort (n = 77). Patients with presumed germline variants are indicated by the solid, blue triangles.

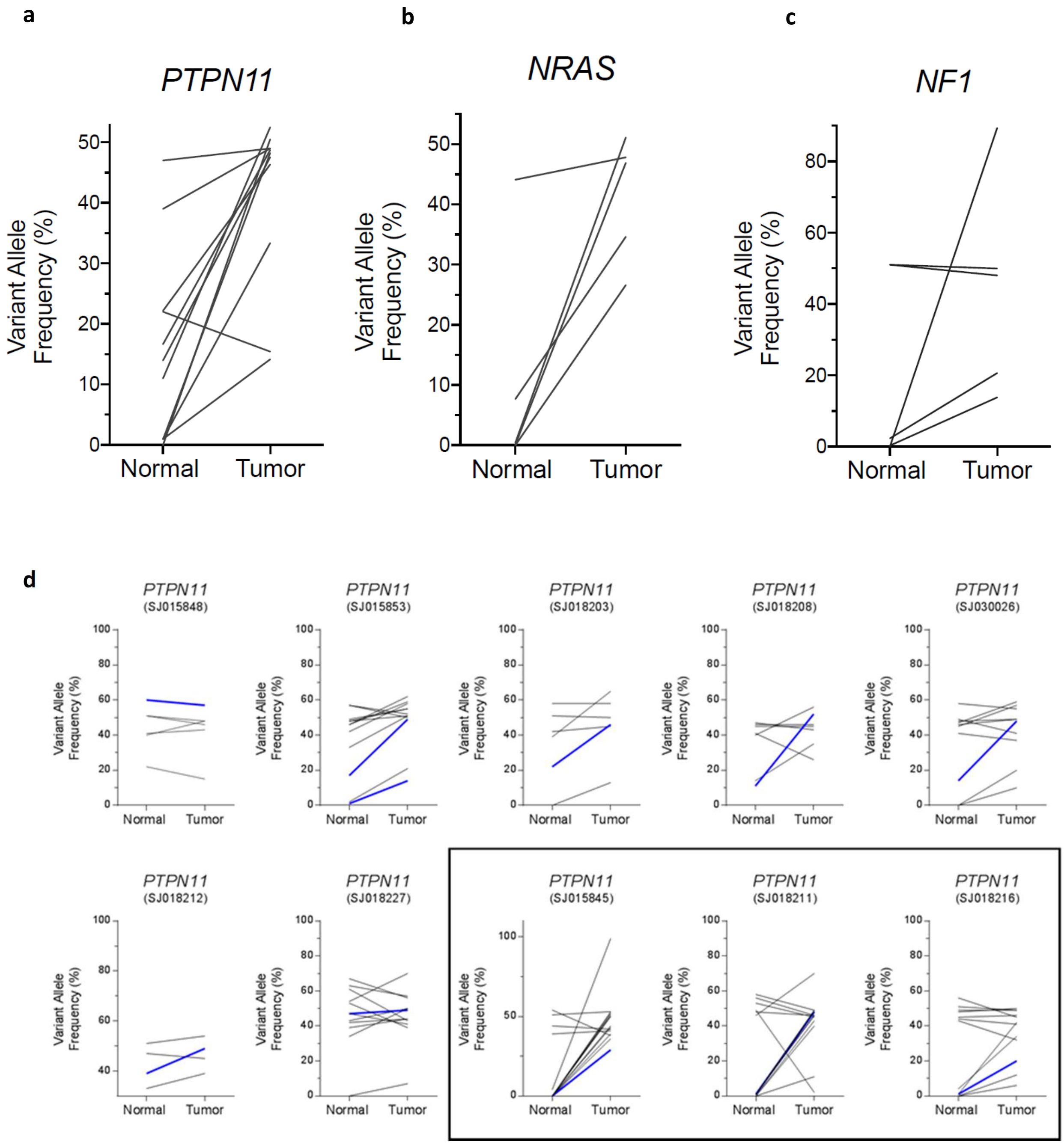

a
PTPN11
Variant Allele Frequency (%)
Normal
Tumor
b
NRAS
Variant Allele Frequency (%)
Normal
Tumor
c
NF1
Variant Allele Frequency (%)
Normal
Tumor
d
PTPN11 (SJ015848)
PTPN11 (SJ015853)
PTPN11 (SJ018203)
PTPN11 (SJ018208)
PTPN11 (SJ030026)
PTPN11 (SJ018212)
PTPN11 (SJ018227)
PTPN11 (SJ015845)
PTPN11 (SJ018211)
PTPN11 (SJ018216)
Variant Allele Frequency (%)
Normal
Tumor

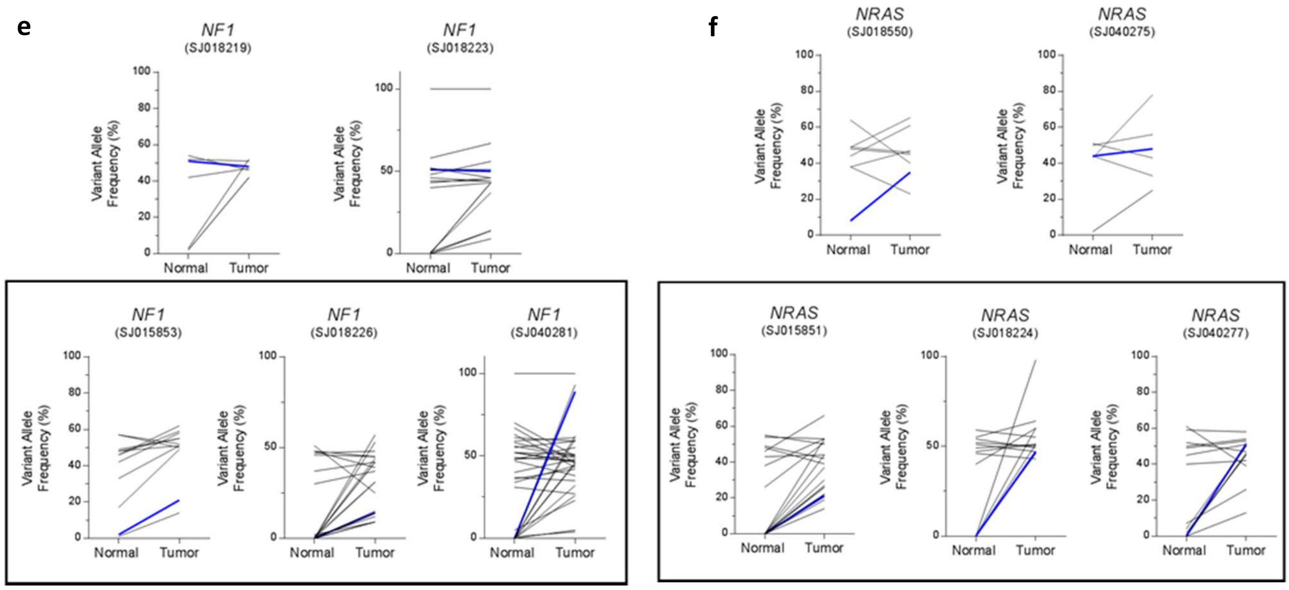

Supplementary Figure 12: Definition of germline variants. VAF plots of patients with *PTPN11* (a), *NRAS* (b), and *NF1* (c) mutations. Only data from WES is shown. In each patient with a *PTPN11*, *NF1*, or *NRAS* mutation in panels a, b, and c, all somatic and presumed germline calls for the individual patients are shown in groups (*PTPN11* (d), *NF1* (e), and *NRAS* (f)). Blue lines indicate the *PTPN11*, *NF1*, and *NRAS* mutations from panels a, b, and c. The plots that are inside the black boxes are those patients in which there is no evidence for the variant in the lymphocytes. Notably, the patients without an obvious deleterious “germline” variant (black boxes) tended to have many more mutations as compared to the cases with evidence of the *PTPN11/NRAS/NF1* variants in the lymphocyte fraction. Further, because the patients with potential germline variants in these important genes tended to have less mutation burden overall (average: 5 mutations/patient for total cohort), it is difficult to determine mosaicism with confidence.

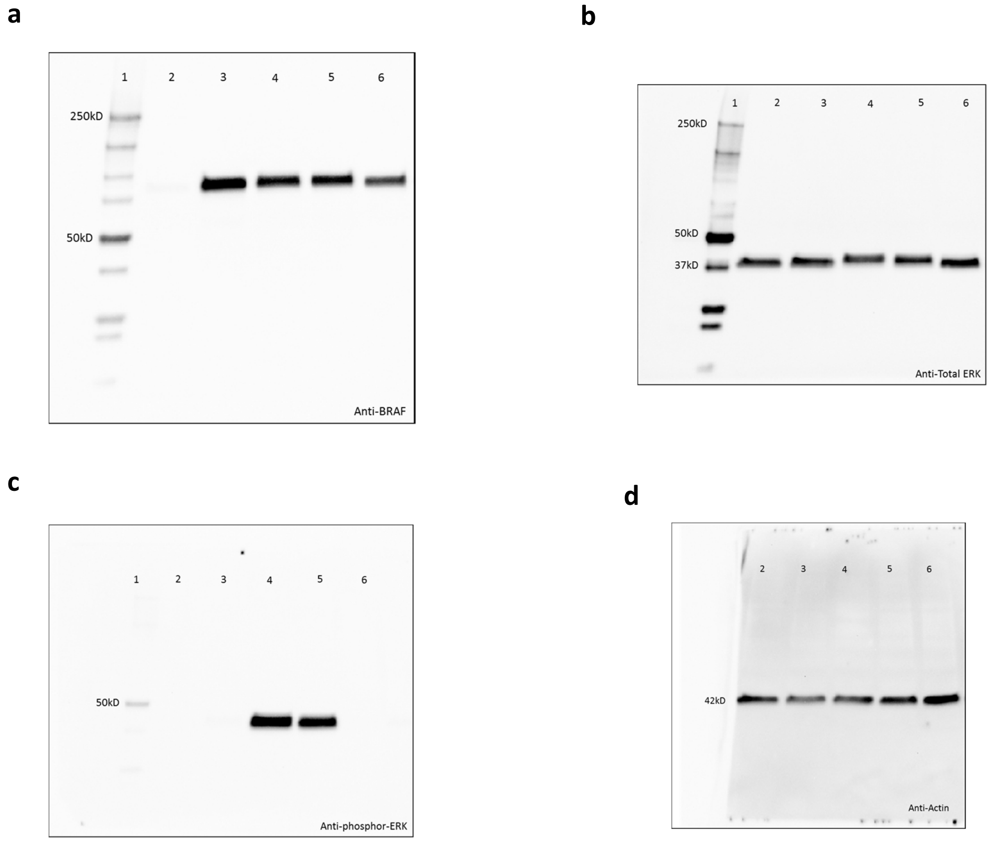

Supplementary Figure 13: Full western blots for transient transfection of *BRAF* mutations in 293T cells. a.) BRAF. b.) Total ERK. C.) phosphorylated ERK. d.) Actin. Lane 1: molecular weight marker, Lane 2: empty vector, Lane 3: BRAF-WT, Lane 4: BRAF-V600E, Lane 5: BRAF-G469A, Lane 6: BRAF-D594N. Note: molecular weight marker was removed from the blot due to substantial differences in chemiluminescence signal between it and the target.

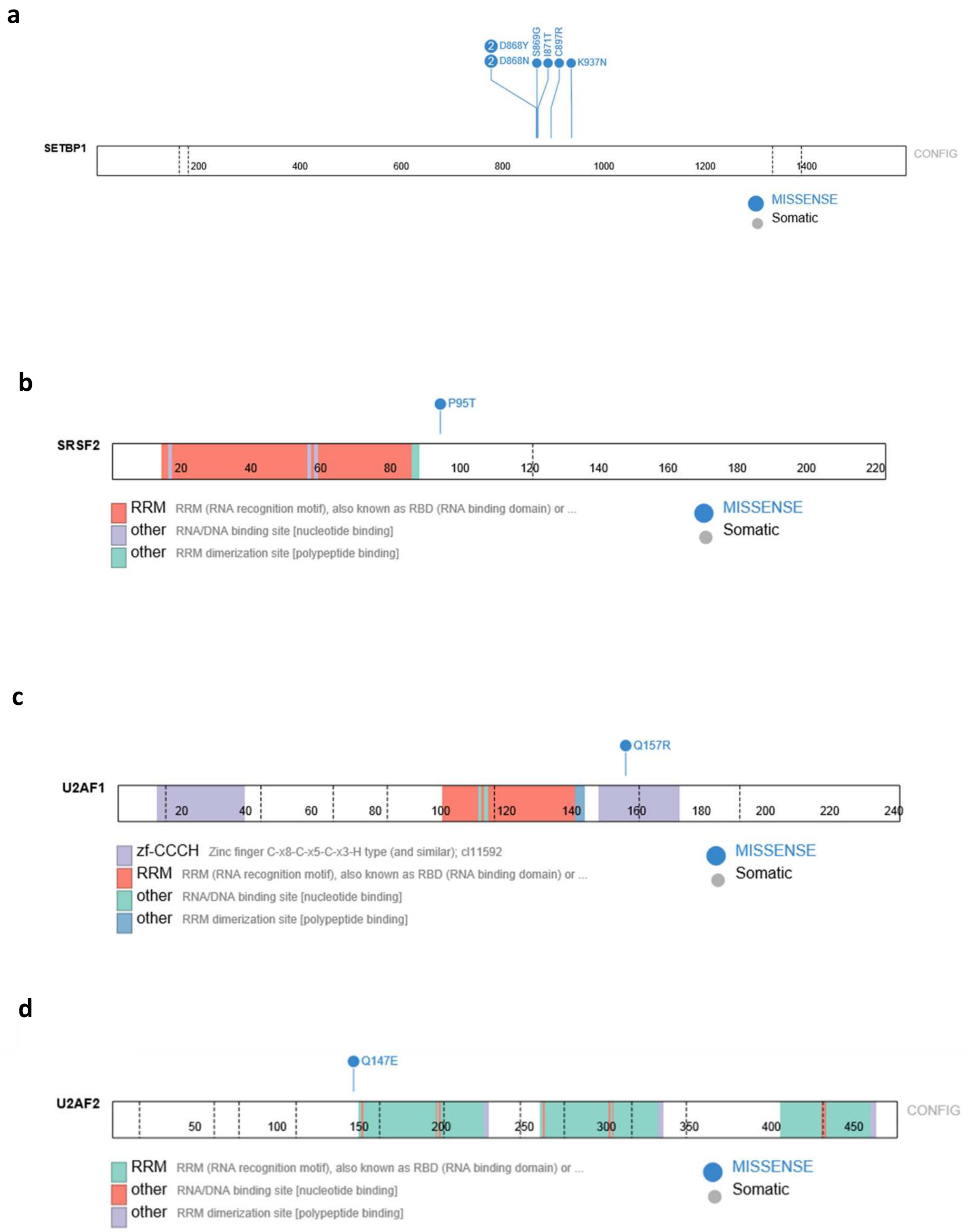

Supplementary Figure 14: Protein Paint schematics showing locations of somatic mutations present in other selected genes in our pediatric primary MDS cohort. *SETBP1* (a), *SRSF2* (b), *U2AF1* (c), *U2AF2* (d).

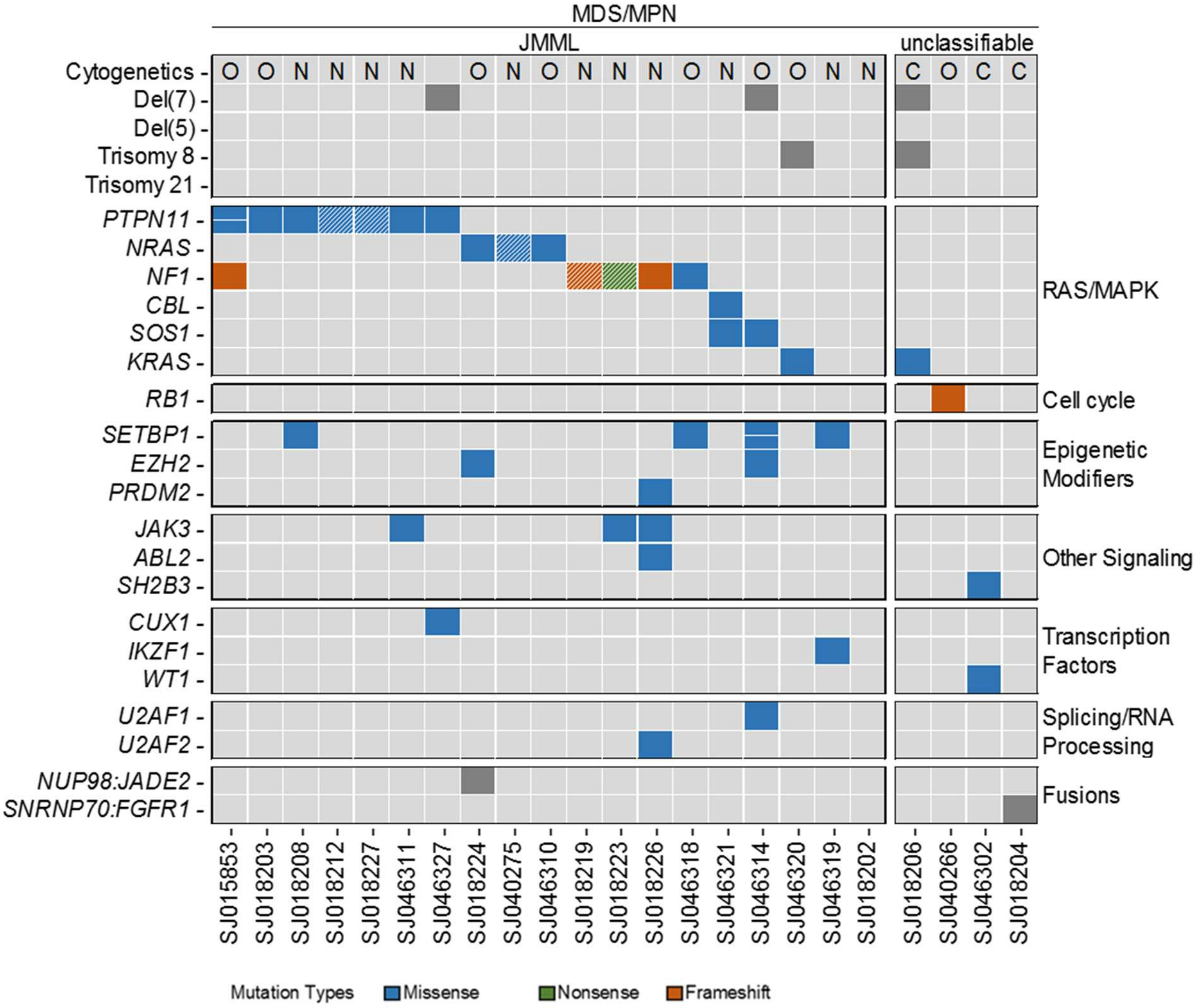

Supplementary Figure 15: Genomic alterations in MDS/MPN. Heat map showing somatic mutations, presumed germline variants (cells with hatched lines), and transcript fusions present in the MDS/MPN cohort (n=23).

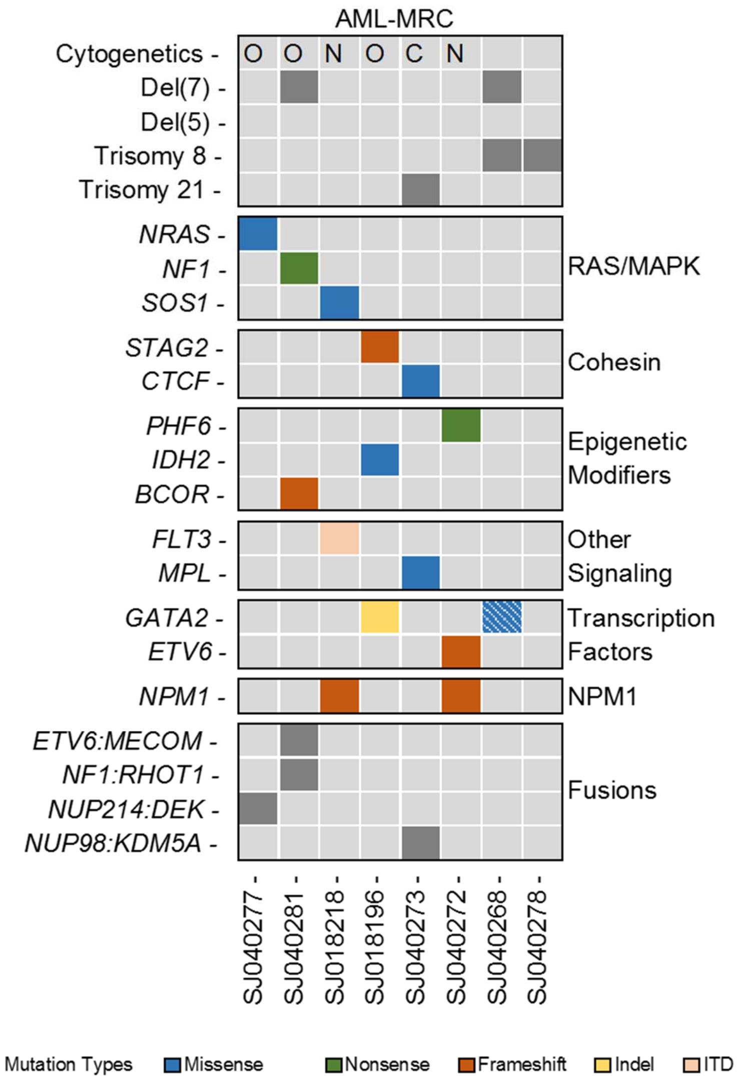

Supplementary Figure 16: Genomic alterations in AML-MRC. Heat map showing somatic mutations, presumed germline variants (cells with hatched lines), and transcript fusions present in the AML-MRC cohort (n=8).

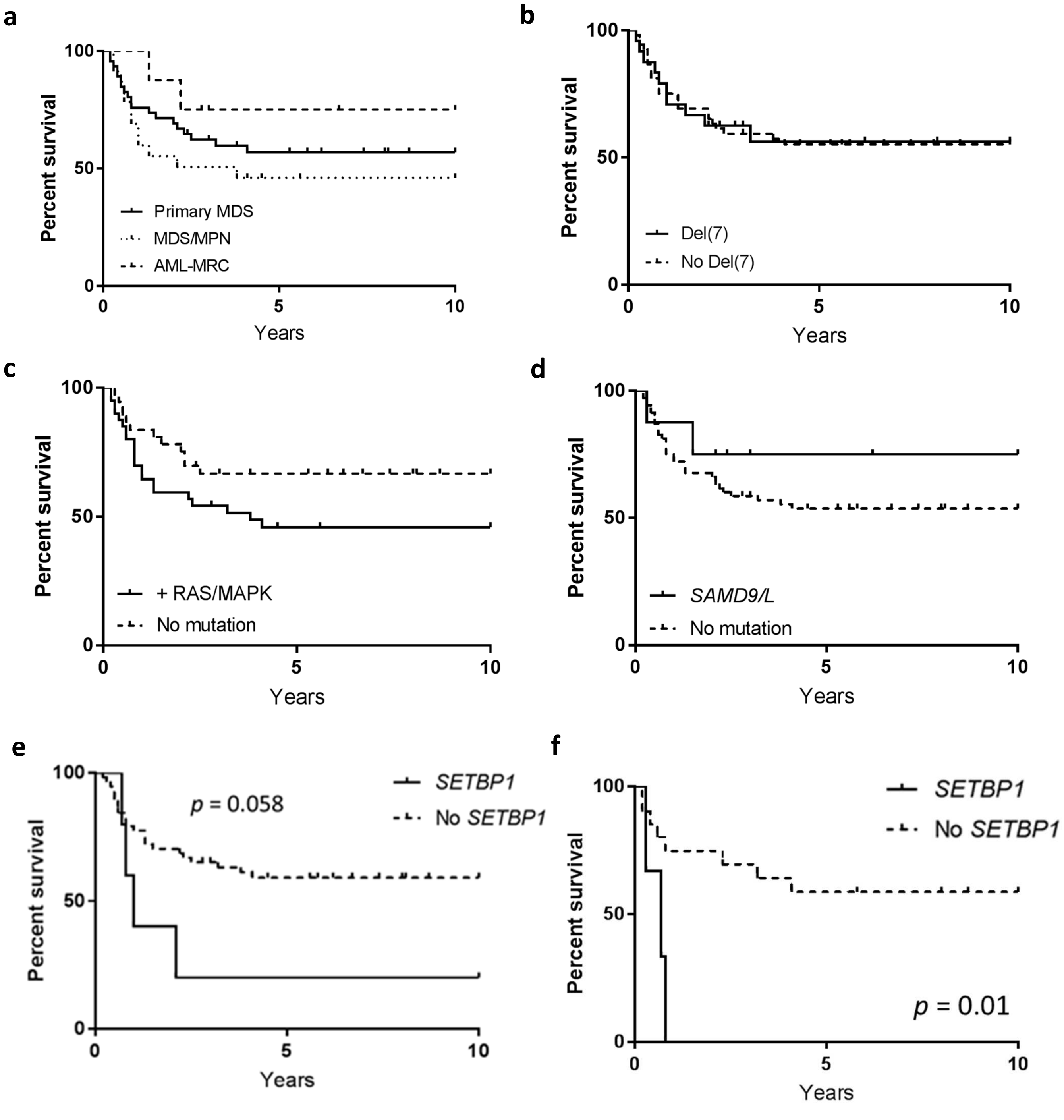

Supplementary Figure 17: Overall survival of pediatric MDS. a-d.) Overall survival is not significantly different between primary MDS, MDS/MPN, and AML-MRC. Survival is also not significantly different if del(7), a RAS/MAPK mutation, or a SAMD9/L mutation is present. e.) Overall survival patients who received a hematopoietic stem cell transplant was not significantly different if a *SETBP1* mutation was present. f.) RAEB patients with a *SETBP1* mutation had a significantly poorer overall survival. Statistical comparisons were made with the log-rank test.

a

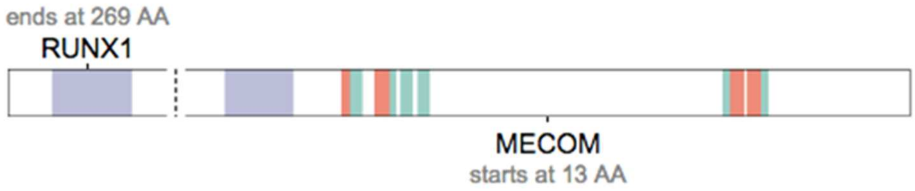

b

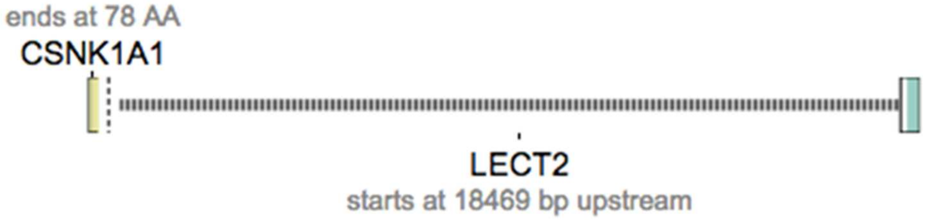

Supplementary Figure 18: Fusion events present in primary MDS: *RUNX1-MEC*OM (a) and *CSNK1A1-LECT2* (b).

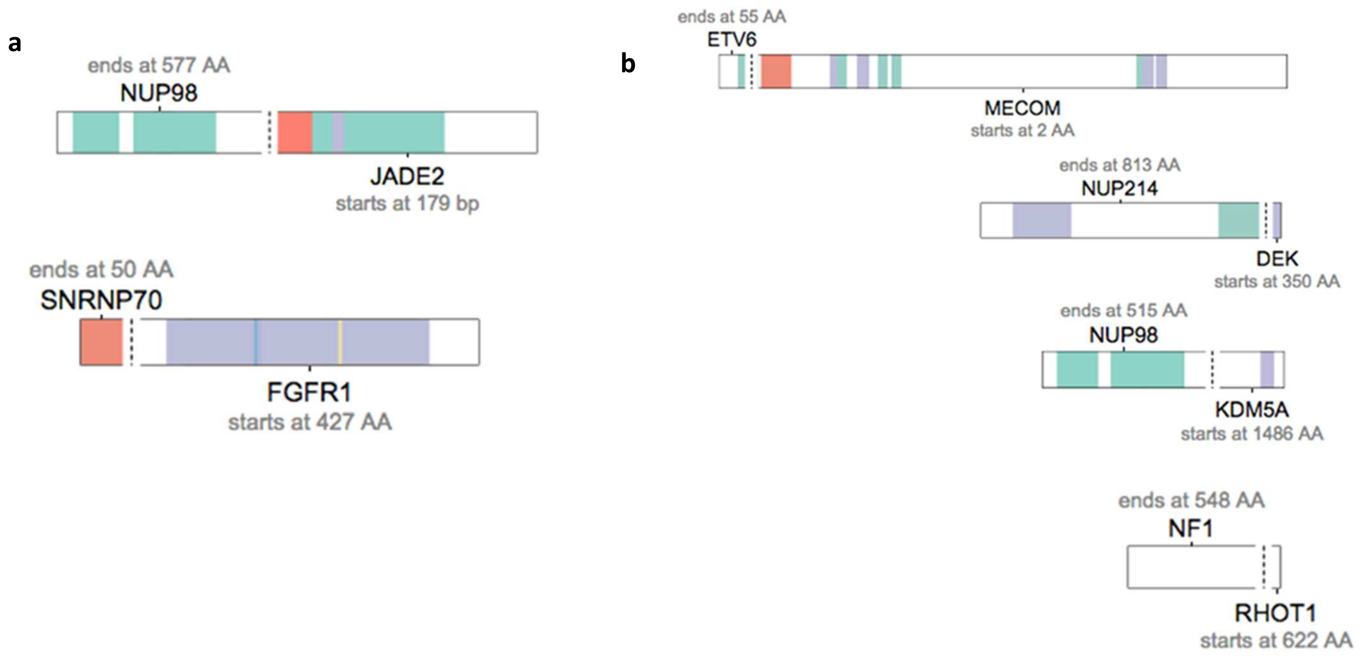

Supplementary Figure 19: Fusion events present in MDS/MPN (a) and AML-MRC (b).

References

1. Alexandrov, L.B. *et al.* Signatures of mutational processes in human cancer. *Nature* **500**, 415-21 (2013).
2. Schwartz, J. *et al.* Germline SAMD9 Mutation in Siblings with Monosomy 7 and Myelodysplastic Syndrome. *Leukemia* **Accepted**(2017).
3. Narumi, S. *et al.* SAMD9 mutations cause a novel multisystem disorder, MIRAGE syndrome, and are associated with loss of chromosome 7. *Nat Genet* **48**, 792-7 (2016).
4. Chen, D.H. *et al.* Ataxia-Pancytopenia Syndrome Is Caused by Missense Mutations in SAMD9L. *Am J Hum Genet* **98**, 1146-58 (2016).
