# Supplementary material for: The genomic landscape of pediatric myelodysplastic syndromes
Source: Nat Commun. 2017 Nov 16;8:1557. doi: 10.1038/s41467-017-01590-5 (PMC5691144; doi:10.1038/s41467-017-01590-5)
Supplement: Supplementary file 3 — Description of Additional Supplementary Files [file 41467_2017_1590_MOESM3_ESM.pdf]

## Description of Additional Supplementary Files

File Name: Supplementary Data 1
Description: Full list of patients within the pediatric MDS cohort including demographic and other clinical laboratory values.

File Name: Supplementary Data 2
Description: Whole exome and RNA sequencing coverage data.

File Name: Supplementary Data 3
Description: Somatic mutation calls from WES, with corresponding validation data.

File Name: Supplementary Data 4
Description: Genes and targets of the TruSeq Custom Amplicon reagent.

File Name: Supplementary Data 5
Description: TruSeq Custom Amplicon coverage data.

File Name: Supplementary Data 6
Description: Mutation calls from TruSeq Custom Amplicon.

File Name: Supplementary Data 7
Description: Copy number analysis (from both conventional karyotyping, and WES, where available) for the entire pediatric MDS cohort.

File Name: Supplementary Data 8
Description: List of genes analyzed for presence of germline alterations.

File Name: Supplementary Data 9
Description: Germline variant calls including ACMG pathogenicity classification for those which were classified.

File Name: Supplementary Data 10
Description: Loss-of-Function mutation calls from WES.

File Name: Supplementary Data 11
Description: *SAMD9/SAMD9L* mutation calls from WES and PCR Validation.

File Name: Supplementary Data 12
Description: Validation primers used for PCR amplification and MiSeq validation.

File Name: Supplementary Data 13
Description: Mutagenesis primers used for creating *BRAF, SAMD9, and SAMD9L* mutations for in vitro studies.
